# Supplementary material for: Prevalence, risk and protective factors of burnout among Korean hospitalists
Source: PLoS One. 2025 Apr 28;20(4):e0320128. doi: 10.1371/journal.pone.0320128 (PMC12036936; doi:10.1371/journal.pone.0320128)
Supplement: S1 File — (DOCX) [file pone.0320128.s001.docx]

**Survey Questionnaire**

**0. Consent to participate in the survey**

Do you agree to participate in this survey (a survey of the current status of hospitalists and factors influencing job satisfaction in Korea)?

□ Yes □ No

**1. Basic information for survey participants**

1-1. Sex: □ Female □ Male

1-2. Age: ( ) years old

1-3. Married: □ Yes □ No

1-4. Do you have children? □ Yes □ No

1-5. What is your year of graduation from medical school? ( ) year

1-6. Please select your trainee department from the following options.

□ Internal medicine □ Surgery □ Family medicine □ Anesthesiology and Pain Medicine □ Radiation Oncology □ Pathology □ Urology □ Obstetrics and Gynecology □ Plastic Surgery □ Pediatrics □ Neurology □ Neurosurgery □ Ophthalmology □ Radiology □ Emergency Medicine □ Otorhinolaryngology □ Rehabilitation Medicine □ Psychiatry and Behavioral Sciences □ Orthopedic Surgery □ Laboratory Medicine □ Dermatology □ Nuclear Medicine □ Cardiothoracic Surgery

1-7. Do you have trainee experience as a clinical instructor (fellowship)?

□ Yes □ No

1-8. What type of fellowship training have you received?

- Internal Medicine □ Infectious Disease □ Endocrinology □ Rheumatology □ Gastroenterology □ Nephrology □ Cardiology □ Hematology-Oncology □ Pulmonology □ Allergy

- Pediatrics □ Pediatric Infectious Disease □ Pediatric Endocrinology □ Pediatric Rheumatology □ Pediatric Gastroenterology □ Pediatric Nephrology □ Pediatric Cardiology □ Pediatric Hematology-Oncology □ Pediatric Pulmonology and Allergy □ Neonatology □ Pediatric Neurology

- Surgery □ Upper Gastrointestinal Surgery □ Colorectal Surgery □ Hepatobiliary Surgery □ Thyroid Surgery □ Breast Surgery

1-9. How many years of experience do you have as a hospitalist?

□ Less than 1 year □ More than 1 year and less than 2 years □ More than 2 years and less than 3 years □ More than 3 years and less than 4 years □ More than 4 years and less than 5 years □ More than 5 years

1-10. What is your current position?

□ Professor □ Associate professor □ Assistant professor □ Clinical professor □ Clinical associate professor □ Clinical assistant professor □ Medical professor □ Other

**2. Basic information about the hospital you work in**

2-1. What type of hospital do you work at?

□ Tertiary general hospital □ General hospital □ Hospital □ Clinic

2-2. Which of the following hospitals do you work in?

□ Public University Hospital □ Private University Hospital □ Public Non-University Hospital □ Private Non-University Hospital

2-3. Please select the size of the hospital where you work.

□ Less than 300 beds □ 300–600 beds □ 600–900 beds

□ 900–1200 beds □ 1200 beds or more

2-4. Where is your work area?

□ Seoul □ Gyeonggi Province (including Incheon)

□ Gyeongsang Province (including Busan, Daegu, and Ulsan)

□ Jeolla Province (including Gwangju) □ Chungcheong Province (including Daejeon and Sejong)

□ Gangwon Province □ Jeju Island

2-5. What is the number of hospitalists in your hospital? ( )

2-6. What is your affiliation within the hospital where you work?

□ Department of Hospital Medicine □ Internal Medicine (General or Comprehensive)

□ Surgery (General or Comprehensive)

□ Specific Specialties (Hematology-Oncology, Neurosurgery, Obstetrics and Gynecology, etc.)

□ Other ( )

**3. Motivation for applying as a hospitalist**

3-1. Financial stability

□ Strongly agree □ Somewhat agree □ Neutral □ Somewhat disagree □ Strongly disagree

3-2. Curiosity and interest in hospital medicine

□ Strongly agree □ Somewhat agree □ Neutral □ Somewhat disagree □ Strongly disagree

3-3. Lack of interest in other fields

□ Strongly agree □ Somewhat agree □ Neutral □ Somewhat disagree □ Strongly disagree

3-4. New opportunity as a hospitalist

□ Strongly agree □ Somewhat agree □ Neutral □ Somewhat disagree □ Strongly disagree

**4. Areas of interest and work areas**

4-1. What are your primary areas of interest within hospital medicine? (Multiple responses)

□ Clinical work □ Education □ Research and evidence-based medicine □ Quality improvement □ None □ Other ( )

4-2. Do you engage in duties outside of clinical practice as a hospitalist? □ Yes □ No

4-3. If you participate in duties outside clinical practice, please select all that apply from the following options:

□ Quality improvement activities □ Hospital committees □ Research □ Education □ Other ( )

4-4. If you participate in duties outside clinical practice, how satisfied are you with these non-clinical responsibilities?

□ Very Satisfied □ Satisfied □ Neutral □ Dissatisfied □ Very Dissatisfied

4-5. Do you believe that your career as a hospitalist aligns with what you expected in terms of clinical practice and non-clinical duties before choosing this path?

□ Very much so □ Yes □ Neutral □ No □ Not at all

4-6. If you answered “No” or “Not at all” to Question 4-5, please provide the reasons for your response.

**5. Education**

5-1. Do you participate in education for staff, residents, or students?

□ Yes □ No

5-2. What is the target audience for your education? (Multiple responses)

□ Hospital staff (physicians, nurses, administrative staff, etc.) □ Residents/interns □ Students

5-3. What is the content of your education? (Multiple responses)

□ Clinical practice (diagnosis, treatment) □ Procedures □ Communication with medical team □ Point-of-care ultrasound □ Other ( )

**6. Clinical practice**

6-1. On average, how many new inpatient admissions (including transfer patients) have you handled in a day over the past month? ( )

6-2. What is the average number of inpatients you’ve treated within the past month? ( )

6-3. What is the maximum number of inpatients you’ve treated within the past month? ( )

6-4. In the past month, what percentage of your inpatient admissions were classified into specialized care disease groups, general care, or simple care categories?

□ Less than 20% □ 20–40% □ 40–60% □ 60–80% □ 80–100%

6-5. How many hours do you work in a week (Monday–Sunday)? [For example, if you work 10 hours a day for 5 days, it would be 50 hours] ( ) hours

6-6. Do you participate in night shifts? □ Yes □ No

6-7. What is the average number of hours you work during the night in a week (between 10 PM and 6 AM)? [For example, if you work approximately 2 hours a day for 3 days, it would be 6 hours] ( ) hours

6-8. What percentage of your duties involve research, staff management, and administrative activities?

□ Less than 20% □ 20–40% □ 40–60% □ 60–80% □ 80–100%

6-9. Does your hospital have physician assistants involved in the care of inpatients managed by hospitalists? □ Yes □ No

6-10. Does your hospital have residents involved in the care of inpatients managed by hospitalists? □ Yes □ No

6-11. What is your work schedule like?

□ 5 days a week (daytime only) □ 7 days a week (daytime only) □ 7 days a week (day and nighttime)

6-12. What is the type of hospital ward where you work?

| - Departmental: Limits inpatient care to specific department patient groups.  - Integrated: Manages complex, critically ill patients collectively.  - Acute: Focuses on resolving issues within 72 hours for patients requiring emergency care.  * Reference: Kim HW. The current status of hospital medicine in Korea, 2019. Korean J Med. 2019;94(2):139–44. |
| --- |

□ Departmental □ Integrated □ Acute

6-13. What is the level of your job authority in clinical care?

□ Very high □ High □ Neutral □ Restricted □ Very restricted

**7. Research**

** Please respond as per research conducted in the last three years (2019–2022).

7-1. Have you been involved in research (academic presentations, paper writing, participation in multi-institutional studies, etc.)?

□ Yes □ No

7-2. Have you received research funding as a principal investigator to conduct research projects in the last three years (including ongoing projects)?

□ Yes □ No

7-3. If you have received research funding and are currently conducting research, which of the following categories best describes your research project? (Multiple responses)

□ Government-funded projects □ Industry-funded projects □ Institutional projects □ Other ( )

7-4. Have you had any experience as the first author (primary author or corresponding author) publishing in academic journals in the last three years? (Multiple responses)

□ None □ Published in SCI/SCIE journals □ Published in SCOPUS journals □ Published in KCI (Korean Citation Index) journals □ Published in other journals

7-5. How many journal articles do you read every week?

□ 0 articles □ 1–5 articles □ 6–10 articles □ 11–15 articles □ 16 or more articles

7-6. Have you attended domestic academic conferences since working as a hospitalist?

□ Yes □ No

7-7. Have you attended international academic conferences since working as a hospitalist?

□ Yes □ No

7-8. Do you have a research mentor?

□ Yes □ No

**8. Satisfaction**

8-1. Are you satisfied as a hospitalist?

□ Very satisfied □ Satisfied □ Neutral □ Dissatisfied □ Very dissatisfied

8-2. Do you think your job satisfaction will improve in the next 5 years compared to now?

□ Very much so □ Yes □ Neutral □ Not really □ Not at all

8-3. Are you satisfied with the time you spend by yourself and with your family while working as a hospitalist?

□ Very satisfied □ Satisfied □ Neutral □ Dissatisfied □ Very dissatisfied

8-4. Are you satisfied with the workload outside regular working hours?

□ Very satisfied □ Satisfied □ Neutral □ Dissatisfied □ Very dissatisfied

8-5. Are you satisfied with the support from the hospital management?

□ Very satisfied □ Satisfied □ Neutral □ Dissatisfied □ Very dissatisfied

8-6. Are you satisfied with the support from the hospitalist leader?

□ Very satisfied □ Satisfied □ Neutral □ Dissatisfied □ Very dissatisfied

8-7. Are you satisfied with your relationships with fellow hospitalists?

□ Very satisfied □ Satisfied □ Neutral □ Dissatisfied □ Very dissatisfied

8-8. Are you satisfied with your relationships with non-hospitalist physicians?

□ Very satisfied □ Satisfied □ Neutral □ Dissatisfied □ Very dissatisfied

8-9. Are you satisfied with your relationships with nurses?

□ Very satisfied □ Satisfied □ Neutral □ Dissatisfied □ Very dissatisfied

**9. Burnout and coping strategies**

9-1. The following questionnaire pertains to self-reporting on burnout. Please indicate how you have felt in the past 6 months. (MBI-HSS)

9-2. What do you consider to be the most suitable methods for teachers to overcome burnout? (Multiple responses)

□ Vacation, travel □ Hobbies □ Self-development □ Job change/resignation
□ Duty-off period □ Other ( )

**10. Mental health problems including depression, anxiety, and stress**

10-1. The following is a self-report questionnaire regarding depression, anxiety, and stress. Please indicate how you have felt in the past month. (DASS-21)

10-2. What factors are associated with your workplace stress? (Multiple responses)

□ Higher severity of patient □ Conflict with caregivers □ Conflict with colleagues

□ Excessive workload □ Long working hours □ Diverse job responsibilities

□ Unnecessary paperwork □ Pressure for achievement □ Low salary

□ Poor working environment □ Other ( )

10-3. Do you believe that leaders in the workplace can be helpful when you experience work-related stress?

□ Very much so □ Yes □ Neutral □ Not really □ Not at all

10-4. Do you believe that medical colleagues and staff in the workplace can provide support when you experience work-related stress?

□ Very much so □ Yes □ Neutral □ Not really □ Not at all

10-5. Do you believe that you can receive support from family and friends when you experience work-related stress?

□ Very much so □ Yes □ Neutral □ Not really □ Not at all

10-6. Do you believe that you can effectively manage stress?

□ Very much so □ Yes □ Neutral □ Not really □ Not at all

10-7. Please select your strategies for stress relief (Multiple responses).

□ Vacation, Travel □ Hobbies □ Self-development

□ Leisure time with family □ Leisure time with friends

10-8. How effective do you consider your stress relief strategies to be?

□ Very effective □ Effective □ Neutral □ Ineffective □ Very ineffective

**11. Insomnia**

11-1. The following is a self-report questionnaire measuring issues related to insomnia. Please indicate how you have felt in the last 2 weeks. (ISI)

**12. Other**

12-1. What is your annual income (after taxes)?

□ Less than 50 million won □ 50–100 million won □ 100–150 million won □ 150–200 million won □ Over 200 million won

12-2. Do you intend to continue working as a hospitalist in the future?

□ Yes □ No □ Not sure

12-3. If you plan to continue working as a hospitalist, what are your reasons? (Select all that apply)

□ Job stability □ Satisfaction with professional expertise □ Work-life balance

□ Other ( )

12-4. If you do not intend to continue working as a hospitalist, what are your reasons? (Select all that apply)

□ Excessive workload □ Salary □ Staff shortage □ Frequent night duty □ Relationship with medical staff □ Job stability □ Social status and recognition □ Lack of sense of accomplishment □ Working environment

□ Other ( )

**13. Please select the most important ones in order of priority for the activation and stability of the hospitalist system.**

□ Improvement of low level of reimbursement to providers

□ Flexibility in hospitalist work regulations (improvement of rigid regulations such as restricted practice to specific wards)

□ Providing various incentives for night, weekend, and holiday shifts, including additional compensation

□ Establishing an independent “Hospital medicine” and introducing a hospitalist training program

□ Ensuring faculty status

□ Other ( )
