# Supplementary material for: Prevalence, risk and protective factors of burnout among Korean hospitalists
Source: PLoS One. 2025 Apr 28;20(4):e0320128. doi: 10.1371/journal.pone.0320128 (PMC12036936; doi:10.1371/journal.pone.0320128)
Supplement: S1 Table — (DOCX) [file pone.0320128.s002.docx]

**Supplementary Table 1.** Work-related characteristics of Korean hospitalists

| **Characteristics** | **Respondents**  **(n=79)** |
| --- | --- |
| Number of Hospitalists per Hospital, median (IQR) | 16 (5–23) |
| Type of work |  |
| Type 1 (daytime only, 5 days a week) | 41 (51.9) |
| Type 2 (daytime only, 7 days a week) | 18 (22.8) |
| Type 3 (day and nighttime, 7 days a week) | 20 (25.3) |
| Mean daily number of new admissions | 4 (3–5) |
| Mean daily number of inpatients | 15 (12–20) |
| Maximum number of inpatients | 19 (15–23) |
| Average working hours in a week | 45 (40–50) |
| Night duty status |  |
| Yes | 28 (35.4) |
| No | 51 (64.6) |
| Night work hours in a week | 14 (9–19.2) |
| Involvement of physician assistant nurses |  |
| Yes | 44 (56.4) |
| No | 34 (43.6) |
| Involvement of residents |  |
| Yes | 15 (19.0) |
| No | 64 (81.0) |
| Autonomy |  |
| High | 48 (60.8) |
| Moderate | 20 (25.3) |
| Low | 11 (13.9) |
| Proportion of non-clinical tasks in addition to patient care. |  |
| 20% or less | 59 (74.7) |
| 20–40% | 17 (21.5) |
| 40–60% | 3 (3.8) |
| Participation in education |  |
| Yes | 59 (74.7) |
| No | 20 (25.3) |
| Research performance |  |
| Yes | 34 (43.0) |
| No | 45 (57.0) |
| Experience with research projects as a principal investigator |  |
| Yes | 14 (17.7) |
| No | 65 (82.3) |
| Participation in academic conference |  |
| Domestic |  |
| Yes | 60 (75.9) |
| No | 19 (24.1) |
| International |  |
| Yes | 16 (20.3) |
| No | 63 (79.7) |
| Number of papers read in a week |  |
| None | 18 (22.8) |
| 1–5 | 56 (70.9) |
| 6–10 | 4 (5.1) |
| Availability of research mentor |  |
| Yes | 14 (17.7) |
| No | 65 (82.3) |

Continuous variables are presented as median and interquartile range (IQR), while categorical variables are expressed as frequency (%)
