# Supplementary material for: Prevalence, risk and protective factors of burnout among Korean hospitalists
Source: PLoS One. 2025 Apr 28;20(4):e0320128. doi: 10.1371/journal.pone.0320128 (PMC12036936; doi:10.1371/journal.pone.0320128)
Supplement: S2 Table — (DOCX) [file pone.0320128.s003.docx]

**Supplemental Table 2.** Correlation analysis between burnout components, DASS, and ISI

| **No.** | **Characteristics** | **1** | **2** | **3** | **4** | **5** | **6** | **7** | **8** | **9** |
| --- | --- | --- | --- | --- | --- | --- | --- | --- | --- | --- |
| 1 | Burnout total | 1 |  |  |  |  |  |  |  |  |
| 2 | Emotional exhaustion | .818^**^ | 1 |  |  |  |  |  |  |  |
| 3 | Depersonalization | .864^**^ | .733^**^ | 1 |  |  |  |  |  |  |
| 4 | Reduced personal accomplishment | -.734^**^ | -.258^*^ | -.444^**^ | 1 |  |  |  |  |  |
| 5 | DASS total | .571^**^ | .523^**^ | .525^**^ | -.334^**^ | 1 |  |  |  |  |
| 6 | Depression | .558^**^ | .535^**^ | .475^**^ | -.338^**^ | .943^**^ | 1 |  |  |  |
| 7 | Anxiety | .477^**^ | .413^**^ | .470^**^ | -.287^*^ | .878^**^ | .718^**^ | 1 |  |  |
| 8 | Stress | .539^**^ | .494^**^ | .515^**^ | -.306^**^ | .964^**^ | .881^**^ | .787^**^ | 1 |  |
| 9 | ISI | .451^**^ | .473^**^ | .420^**^ | -0.203 | .500^**^ | .387^**^ | .552^**^ | .469^**^ | 1 |

^*^ P value < 0.05; ^**^ P value < 0.01
