# Supplementary material for: Prevalence, risk and protective factors of burnout among Korean hospitalists
Source: PLoS One. 2025 Apr 28;20(4):e0320128. doi: 10.1371/journal.pone.0320128 (PMC12036936; doi:10.1371/journal.pone.0320128)
Supplement: S3 Table — (DOCX) [file pone.0320128.s004.docx]

**Supplementary Table 3.** Subjective reported factors related to burnout of the Korean hospitalists

| **Characteristics** | **Emotional exhaustion** | | | **Depersonalization** | | | **Reduced personal accomplishment** | | |
| --- | --- | --- | --- | --- | --- | --- | --- | --- | --- |
|  | **Low (n=59)** | **Moderate (n=12)** | **High  (n=8)** | **Low (n=21)** | **Moderate (n=18)** | **High  (n=40)** | **Low (n=10)** | **Moderate (n=24)** | **High  (n=45)** |
| Higher severity of patient | 30 (50.9) | 3 (2.5) | 7 (88.0) | 9 (42.9) | 13 (72.2) | 18 (4.5) | 5 (50.0) | 13 (54.2) | 22 (48.9) |
| Conflict with caregivers | 25 (42.4) | 4 (33.3) | 3 (37.5) | 6 (28.6) | 7 (38.9) | 19 (47.5) | 3 (30.0) | 7 (29.2) | 22 (48.9) |
| Conflict with colleagues | 15 (25.4) | 2 (16.7) | 3 (37.5) | 7 (33.3) | 2 (11.1) | 11 (27.5) | 0 (0) | 9 (37.5) | 11 (24.4) |
| Excessive workload | 16 (27.1) | 3 (2.5) | 4 (50.0) | 0 (0.0) | 6 (33.3) | 17 (42.5) | 4 (40.0) | 6 (25.0) | 13 (28.9) |
| Long working hours | 10 (17.0) | 5 (41.7) | 5 (62.5) | 3 (14.3) | 0 (0) | 17 (42.5) | 2 (20.0) | 5 (20.8) | 13 (28.9) |
| Diverse job responsibilities | 7 (11.9) | 1 (8.3) | 2 (25.0) | 0 (0.0) | 5 (27.8) | 5 (12.5) | 2 (20.0) | 2 (8.3) | 6 (13.3) |
| Unnecessary paperwork | 10 (17.0) | 2 (16.7) | 3 (37.5) | 2 (9.5) | 1 (5.6) | 12 (30.0) | 1 (10.0) | 5 (20.8) | 9 (20.0) |
| Pressure for achievement | 9 (15.3) | 2 (16.7) | 2 (25.0) | 3 (14.3) | 2 (11.1) | 8 (20.0) | 1 (10.0) | 4 (16.7) | 8 (17.8) |
| Low salary | 12 (20.3) | 3 (2.5) | 6 (75.0) | 7 (33.3) | 2 (11.1) | 12 (30.0) | 5 (50.0) | 5 (20.8) | 11 (24.4) |
| Poor working environment | 8 (13.6) | 3 (2.5) | 3 (37.5) | 4 (19.1) | 1 (5.6) | 9 (22.5) | 3 (30.0) | 2 (8.3) | 9 (20.0) |

Data are expressed as frequency (%).
