# Supplementary material for: Prevalence, risk and protective factors of burnout among Korean hospitalists
Source: PLoS One. 2025 Apr 28;20(4):e0320128. doi: 10.1371/journal.pone.0320128 (PMC12036936; doi:10.1371/journal.pone.0320128)
Supplement: S1 Fig — (DOCX) [file pone.0320128.s005.docx]

**Supplementary Figure 1.** Correlation between the burnout symptoms and the number of hospitalists

| 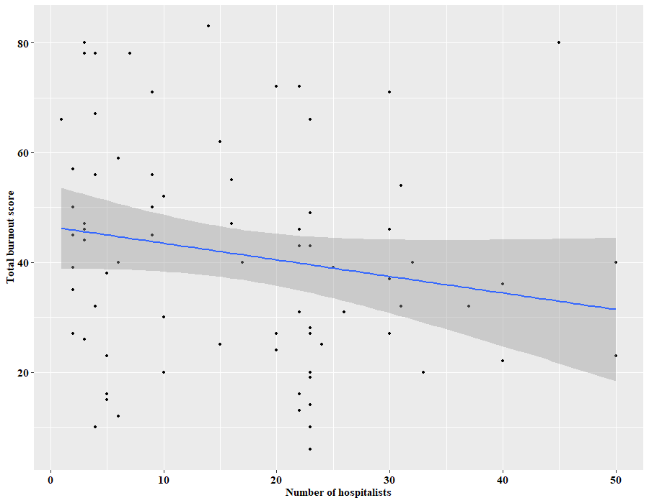   1. Correlation between total burnout score and the number of hospitalists | 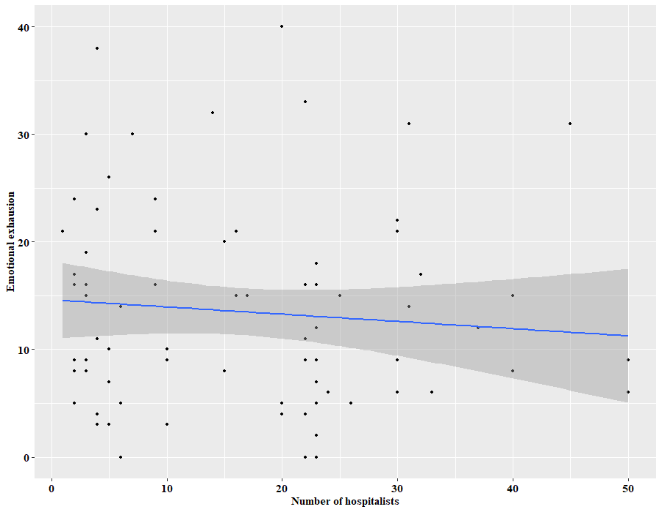   1. Correlation between emotional exhaustion score and the number of hospitalists |
| --- | --- |
| 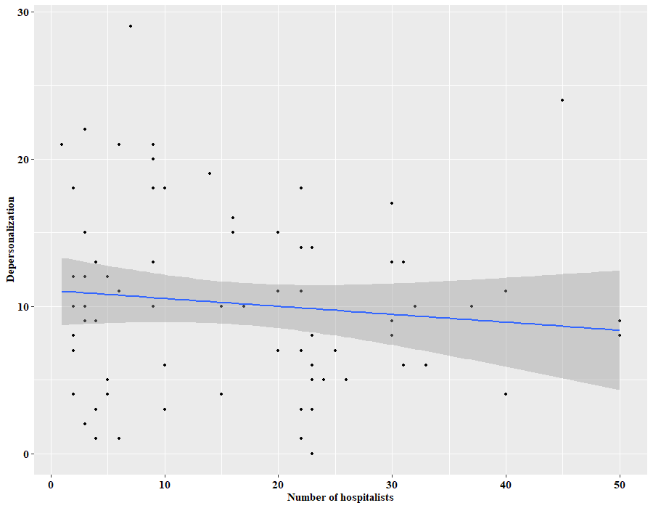   1. Correlation between depersonalization score and the number of hospitalists | 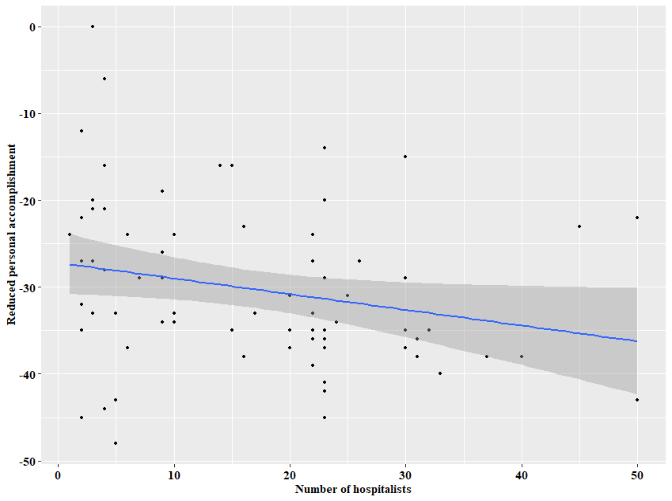   1. Correlation between reduced personal accomplishment score and the number of hospitalists |

Correlation between burnout symptoms and number of hospitalists A. Correlation between total burnout score and number of hospitalists; B. Correlation between emotional exhaustion score and number of hospitalists; C. Correlation between depersonalization score and number of hospitalists; D Correlation between reduced personal accomplishment score and number of hospitalists.
