# Supplementary material for: Prevalence, risk and protective factors of burnout among Korean hospitalists
Source: PLoS One. 2025 Apr 28;20(4):e0320128. doi: 10.1371/journal.pone.0320128 (PMC12036936; doi:10.1371/journal.pone.0320128)
Supplement: Attachment — Submitted filename: reviewer comment_20241212.docx [file pone.0320128.s007.docx]

**Reviewer #1: Greetings, based on my review, the document complies with the requirements for publication. However, it is recommended to specify the study approach, whether it is qualitative or quantitative, the type of design (experimental or non-experimental), and the sampling method (probabilistic or non-probabilistic: simple random, convenience, etc.). Additionally, it is important to specify the selection criteria according to the study and the reasons behind this selection.**

Response: Thank you for your valuable feedback. This study is a quantitative, non-experimental study designed to assess burnout and its associated factors among hospitalists. The study included all hospitalists registered as members of the Korean Association of Internal Medicine and Korean Society of Hospital Medicine (n=303) out of 384 registered Korean hospitalists at the time of the survey. This information has been added to the Methods section for greater clarity. We revised the manuscript as follows.

<Material and Methods, Study design and target population, First paragraph>

From: The study was a cross-sectional survey conducted over 20 days, from January 30 to February 18, 2023. The target population comprised all hospitalists in Korea (n=303).

To: The study was a cross-sectional survey conducted over 20 days, from January 30 to February 18, 2023. Among 384 registered Korean hospitalists as of February 2023, the target population comprised 303 hospitalists who were members of the Korean Association of Internal Medicine and Korean Society of Hospital Medicine.

**Reviewer #2: Thank you for the opportunity to review, here are my comments:**

**1) p. 4 line 54 should be modified "can potentially cause stress and eventual burnout"**

Response: Thank you for pointing out this wording. We agree that it can be improved for clarity. The phrase has been modified to more accurately convey the progression from stress to burnout.

<Introduction, Second paragraph>

From: Despite the benefits of introducing hospitalists to hospitals, focusing solely on their productivity can potentially cause burnout and stress.

To: Despite the benefits of introducing hospitalists to hospitals, focusing solely on their productivity can potentially cause stress and eventual burnout.

**2) p. 3 lines 53-63: Just reading this should show any reader that there is a major problem in how burnout prevalence is assessed. 17.6% to 76% is a ridiculous range.**

**Rotenstein, L. S., Torre, M., Ramos, M. A., Rosales, R. C., Guille, C., Sen, S., & Mata, D. A. (2018). Prevalence of burnout among physicians: a systematic review. Jama, 320(11), 1131-1150.**

Response: Thank you for highlighting this concern regarding the wide range in reported burnout prevalence. As you noted, the range of 17.6% to 76% reflects significant variability in assessment methods, measurement tools, study populations, and burnout definitions across studies. We have revised this section to provide better context and added a citation to Rotenstein et al. (2018) to underscore the issue of inconsistent burnout assessment methods in existing literature.

<Introduction, Second paragraph>

From: Medical professionals are vulnerable to burnout. According to a review of burnout among medical residents, the burnout rate varied from 17.6% to 76.0%. [9] Another study claimed that burnout among doctors ranged from 12% to 61%. [10]

To: "Medical professionals are vulnerable to burnout. Although interpretation requires caution due to differences in assessment methods, measurement tools, and study populations [9], according to a review of burnout among medical residents, the burnout rate varied from 17.6% to 76.0%. [10] Another study claimed that burnout among doctors ranged from 12% to 61%. [11]"

Ref.

[9] Rotenstein LS, Torre M, Ramos MA, et al. Prevalence of Burnout Among Physicians: A Systematic Review. JAMA. 2018;320(11):1131-1150. doi:10.1001/jama.2018.12777

**3) p. 4 line 83: Are there only 303 hospitalists in Korea? Or was this the sample you ended up with after inviting all hospitalists to participate?**

Response: Thank you for seeking clarification. At the time of the study, there were 384 hospitalists actively working in Korea. The study included all hospitalists registered as members of the Korean Association of Internal Medicine and Korean Society of Hospital Medicine (n=303) out of 384 registered Korean hospitalists at the time of the survey. We sent an online survey link to these members through e-mail and text messages. We have clarified this in the manuscript to avoid any ambiguity.

<Material and Methods, Study design and target population, First paragraph>

From: The study was a cross-sectional survey conducted over 20 days, from January 30 to February 18, 2023. The target population comprised all hospitalists in Korea (n=303). The online survey link was sent to the participants through e-mail and text messages by the Korean Association of Internal Medicine and Korean Society of Hospital Medicine.

To: The study was a cross-sectional survey conducted over 20 days, from January 30 to February 18, 2023. Among 384 registered Korean hospitalists, the target population comprised 303 hospitalists who were members of the Korean Association of Internal Medicine and Korean Society of Hospital Medicine. The online survey link was sent through e-mail and text messages.

**4) Provide a correlation table with burnout, burnout components, DASS components and Insomnia index and comment on the overlaps present between the variables in your study.**

Response: We present a correlation table between the total burnout score and its components, the total DASS score and its components, and the Insomnia Index.

| Supplemental Table 1. Correlation analysis between burnout components, DASS, and ISI | | | | | | | | | | |
| --- | --- | --- | --- | --- | --- | --- | --- | --- | --- | --- |
| **No.** | **Characteristics** | **1** | **2** | **3** | **4** | **5** | **6** | **7** | **8** | **9** |
| 1 | Burnout total | 1 |  |  |  |  |  |  |  |  |
| 2 | Emotional exhaustion | .818^**^ | 1 |  |  |  |  |  |  |  |
| 3 | Depersonalization | .864^**^ | .733^**^ | 1 |  |  |  |  |  |  |
| 4 | Reduced personal accomplishment | -.734^**^ | -.258^*^ | -.444^**^ | 1 |  |  |  |  |  |
| 5 | DASS total | .571^**^ | .523^**^ | .525^**^ | -.334^**^ | 1 |  |  |  |  |
| 6 | Depression | .558^**^ | .535^**^ | .475^**^ | -.338^**^ | .943^**^ | 1 |  |  |  |
| 7 | Anxiety | .477^**^ | .413^**^ | .470^**^ | -.287^*^ | .878^**^ | .718^**^ | 1 |  |  |
| 8 | Stress | .539^**^ | .494^**^ | .515^**^ | -.306^**^ | .964^**^ | .881^**^ | .787^**^ | 1 |  |
| 9 | ISI | .451^**^ | .473^**^ | .420^**^ | -0.203 | .500^**^ | .387^**^ | .552^**^ | .469^**^ | 1 |

^*^ P value < 0.05; ^**^ P value < 0.01

The correlation analysis revealed statistically significant relationships between the burnout total score and burnout components, DASS components, and the Insomnia Index, with the exception of Reduced Personal Accomplishment and ISI. Among the DASS components—Depression, Anxiety, and Stress—the correlation was strongest between the Burnout total score and Depression (0.558, p<0.01), followed by Stress (0.539, p<0.01) and Anxiety (0.477, p<0.01). When examining individual burnout components, we found that Emotional Exhaustion, Depersonalization, and Reduced Personal Accomplishment all showed the highest correlation with Depression. For the Insomnia Index, the highest correlation was observed with Emotional Exhaustion.

<Result, Burnout and psychiatric symptoms among hospitalist, First paragraph>

From: More than half of the respondents screened positively for two burnout domains: 50.6% (n=40) for depersonalization, 57.0% (n=45) for reduced personal accomplishment, and 10.1% (n=8) reported high degree of emotional exhaustion (Figure 1). Of the respondents, 7.5% (n=6) reported mild to moderate depressive symptoms and 5.1% (n=4) reported mild to moderate anxiety symptoms. None of the respondents screened positive for severe depression, anxiety, or stress. Approximately 30.4% (n=24) of the respondents reported experiencing subthreshold insomnia and 8.9% (n=7) were screened for moderate to severe insomnia (Table 2).

To: More than half of the respondents screened positively for two burnout domains: 50.6% (n=40) for depersonalization, 57.0% (n=45) for reduced personal accomplishment, and 10.1% (n=8) reported high degree of emotional exhaustion (Figure 1). Of the respondents, 7.5% (n=6) reported mild to moderate depressive symptoms and 5.1% (n=4) reported mild to moderate anxiety symptoms. None of the respondents screened positive for severe depression, anxiety, or stress. Approximately 30.4% (n=24) of the respondents reported experiencing subthreshold insomnia and 8.9% (n=7) were screened for moderate to severe insomnia (Table 2). An analysis of the correlations between each burnout component, DASS, and ISI revealed statistically significant correlations, with the exception of the correlation between Reduced Personal Accomplishment and ISI (Supplemental Table 1).

**5) Exhaustion is the main component of burnout, and burnout is a syndrome, so why focus only on depersonalization and personal accomplishment. Comment on the exhaustion levels.**

**Canu, I. G., Marca, S. C., Dell’Oro, F., Balázs, Á., Bergamaschi, E., Besse, C., ... & Wahlen, A. (2021). Harmonized definition of occupational burnout: A systematic review, semantic analysis, and Delphi consensus in 29 countries. Scandinavian journal of work, environment & health, 47(2), 95.**

**Schaufeli, W. (2021). The burnout enigma solved?. Scandinavian journal of work, environment & health, 47(3), 169.**

Response: Thank you for your suggestion. It was not our intention to exclude the emotional exhaustion domain. However, in our study, the severity of the other two domains (depersonalization and reduced personal accomplishment) were measured to be higher than that of emotional exhaustion, and in emphasizing this aspect, our wording may have led to some misunderstanding. We have revised this section as follows.

<Result, Burnout and psychiatric symptoms among hospitalist, First paragraph>

From: More than half of the respondents screened positively for two burnout domains: depersonalization and reduced personal accomplishment (Figure 1).

To: More than half of the respondents screened positively for two burnout domains: 50.6% (n=40) for depersonalization, 57.0% (n=45) for reduced personal accomplishment, and 10.1% (n=8) reported high degree of emotional exhaustion (Figure 1).

**6) Again, in your discussion you say that your burnout prevalence is approximately 50%. This is not accurate, burnout cannot be diagnosed. You are only estimating risk. So if anything you are estimating burnout risk prevalence, not burnout itself. This should be emphasized. Again, I am somewhat taken aback that exhaustion is not considered here.**

**Maslach, C., & Leiter, M. P. (2021). How to measure burnout accurately and ethically. Harvard Business Review, 7. https://hbr.org/2021/03/how-to-measure-burnout-accurately-and-ethically**

Response: Thank you for your considerate comment. As you pointed out, since we used the MBI-HSS to measure evaluate burnout, it would be more appropriate to consider it as assessing risk. In addition, we added comment about the result of emotional exhaustion of this study. The revised manuscript is as follows.

<Discussion, Second paragraph>

From: The prevalence of burnout among hospitalists in our study (approximately 50%) closely aligns with that found in previous research (30% to 60%), [11-13] including the findings in Roberts et al. (52.3% among 130 internal-medicine hospitalists). [13] The difference from previous studies lies in the fact that, while they focused on internal medicine hospitalists, we targeted hospitalists with diverse specialties. Despite this distinction, the similarity in the findings suggests a substantial burnout rate among hospitalists, irrespective of their specialization. In a previous study, high levels of emotional exhaustion and depersonalization were reported, [13] whereas in the present study, elevated levels of depersonalization and reduced personal accomplishment were observed. Emotional exhaustion tends to be strongly associated with job demand variables, including work overload, whereas depersonalization and personal accomplishment are more associated with resources, including social support or the quality of job contents. [25] This indicates that burnout among Korean hospitalists may be influenced more by the quality of their work than by the quantity of their workload.

To: The signs of burnout among hospitalists in our study, which could be lead to higher risk for burnout in Korean hospitalists, closely aligns with that found in previous research (30% to 60%), [11-13] including the findings in Roberts et al. (52.3% among 130 internal-medicine hospitalists). [13] The difference from previous studies lies in the fact that, while they focused on internal medicine hospitalists, we targeted hospitalists with diverse specialties. Despite this distinction, the similarity in the findings suggests a substantial burnout rate among hospitalists, irrespective of their specialization. In a previous study, high levels of emotional exhaustion and depersonalization were reported, [13] whereas in the present study, elevated levels of depersonalization and reduced personal accomplishment with relatively low levels of emotional exhaustion were observed. Depersonalization and personal accomplishment are more associated with resources, including social support or the quality of job contents, while emotional exhaustion tends to be strongly associated with job demand variables, including work overload. [25] Thus, the result of this study indicates that burnout among Korean hospitalists may be influenced more by the quality of their work than by the quantity of their workload.

**7) It would also be interesting to see a cross-tabulation of your burnout risk categories with the DASS categories.**

Response: Thank you for highlighting points that add interest to this study. By comparing burnout risk categories with DASS categories, we can create a cross-tabulation table that yields the following results.

| Supplemental Table 2. Distribution of DASS components according to burnout risk components | | | | | | | | | | | |
| --- | --- | --- | --- | --- | --- | --- | --- | --- | --- | --- | --- |
| **Component** | **Category** | | **Depression** | | | **Anxiety** | | | **Stress** | | |
|  |  |  | **Normal** | **Mild** | **Moderate** | **Normal** | **Mild** | **Moderate** | **Normal** | **Mild** | **Moderate** |
| Emotional exhaustion | Low | N | 55 | 4 | 0 | 57 | 0 | 1 | 59 | 0 | 0 |
|  |  | % | 93.2% | 6.8% | 0.0% | 98.3% | 0.0% | 1.7% | 100.0% | 0.0% | 0.0% |
|  | Moderate | N | 12 | 0 | 0 | 12 | 0 | 0 | 11 | 0 | 0 |
|  |  | % | 100.0% | 0.0% | 0.0% | 100.0% | 0.0% | 0.0% | 100.0% | 0.0% | 0.0% |
|  | High | N | 6 | 1 | 1 | 5 | 3 | 0 | 8 | 0 | 0 |
|  |  | % | 75.0% | 12.5% | 12.5% | 62.5% | 37.5% | 0.0% | 100.0% | 0.0% | 0.0% |
|  |  |  | P value = 0.111 | | | P value = 0.002 | | | P value = NA | | |
| Depersonalization | Low | N | 20 | 1 | 0 | 20 | 0 | 0 | 21 | 0 | 0 |
|  |  | % | 95.2% | 4.8% | 0.0% | 100.0% | 0.0% | 0.0% | 100.0% | 0.0% | 0.0% |
|  | Moderate | N | 18 | 0 | 0 | 18 | 0 | 0 | 18 | 0 | 0 |
|  |  | % | 100.0% | 0.0% | 0.0% | 100.0% | 0.0% | 0.0% | 100.0% | 0.0% | 0.0% |
|  | High | N | 35 | 4 | 1 | 36 | 3 | 1 | 39 | 0 | 0 |
|  |  | % | 87.5% | 10.0% | 2.5% | 90.0% | 7.5% | 2.5% | 100.0% | 0.0% | 0.0% |
|  |  |  | P value = 0.702 | | | P value = 0.704 | | | P value = NA | | |
| Reduced personal accomplishment | Low | N | 10 | 0 | 0 | 10 | 0 | 0 | 10 | 0 | 0 |
|  |  | % | 100.0% | 0.0% | 0.0% | 100.0% | 0.0% | 0.0% | 100.0% | 0.0% | 0.0% |
|  | Moderate | N | 24 | 0 | 0 | 23 | 0 | 0 | 24 | 0 | 0 |
|  |  | % | 100.0% | 0.0% | 0.0% | 100.0% | 0.0% | 0.0% | 100.0% | 0.0% | 0.0% |
|  | High | N | 39 | 5 | 1 | 41 | 3 | 1 | 44 | 0 | 0 |
|  |  | % | 86.7% | 11.1% | 2.2% | 91.1% | 6.7% | 2.2% | 100.0% | 0.0% | 0.0% |
|  |  |  | P value = 0.385 | | | P value = 0.828 | | | P value = NA | | |

* Fisher’s exact test was applied for all statistical testing due to more than 20% of cells having an expected frequency of less than 5

* NA: Not Applicable

Overall, when the burnout component was in the 'High' group, a higher proportion of participants fell into the Mild or Moderate categories of the DASS components. For Emotional Exhaustion in the 'High' group, 12.5% were in both the Mild and Moderate Depression groups, and 37.5% were in the Mild Anxiety group. For Depersonalization in the 'High' group, 10% were in the Mild Depression group, 2.5% in the Moderate Depression group, 7.5% in the Mild Anxiety group, and 2.5% in the Moderate Anxiety group. Finally, for Reduced Personal Accomplishment in the 'High' group, 11.1% were in the Mild Depression group, 2.2% in the Moderate Depression group, 6.7% in the Mild Anxiety group, and 2.2% in the Moderate Anxiety group. No participants were classified in the Mild or Moderate groups for the Stress component of the DASS.

Statistical analysis revealed no significant differences overall between burnout components and each DASS component; however, a statistically significant difference was observed between Emotional Exhaustion and Anxiety (p-value = 0.002).

<Results, Burnout and psychiatric symptoms among hospitalists, First paragraph>

From: Approximately 30.4% (n=24) of the respondents reported experiencing subthreshold insomnia and 8.9% (n=7) were screened for moderate to severe insomnia (Table 2). An analysis of the correlations between each burnout component, DASS, and ISI revealed statistically significant correlations, with the exception of the correlation between Reduced Personal Accomplishment and ISI. (Supplemental Table 1)

To: Approximately 30.4% (n=24) of the respondents reported experiencing subthreshold insomnia and 8.9% (n=7) were screened for moderate to severe insomnia (Table 2). An analysis of the correlations between each burnout component, DASS, and ISI revealed statistically significant correlations, with the exception of the correlation between Reduced Personal Accomplishment and ISI. (Supplemental Table 1) Overall, when the burnout component was in the 'High' group, the proportion of participants in the Mild or Moderate categories of the DASS components was relatively higher (Supplemental Table 2).

6. PLOS authors have the option to publish the peer review history of their article (what does this mean?). If published, this will include your full peer review and any attached files.

Other comments:

**- In Table 1, it is unclear what the p-values refer to, as no comparisons are explicitly mentioned. Additionally, it would be helpful to compare the characteristics of respondents with those of the full sample of 303 hospitalists to whom the questionnaire was sent. This comparison could provide insight into any potential response bias and the representativeness of the study sample.**

Response: Thank you for providing comments that help in understanding the study. As mentioned, the p-values in Table 1 were excluded due to their potential to cause confusion. These p-values were derived from statistical analyses where the burnout total score was the dependent variable, and each variable listed in Table 1: Characteristics was an independent variable. However, as these values appeared to be information that might disrupt the overall flow of the study, the decision was made to exclude them.

To analyze nonresponse bias, we conducted the following analysis based on previous literature. Responses were collected over a total of 16 days, with the distribution of responses by date as follows: 26, 10, 7, 8, 5, 1, 1, 7, 1, 1, 1, 6, 0, 0, 3, 1, 0, and 1. Assuming that the responses of nonrespondents would most closely resemble those of late responders, we defined early responders as those who responded within the first five days and late responders as those who responded thereafter. The results of statistical tests comparing the two groups showed no statistically significant differences across all variables.

Ref.

Johnson TP, Wislar JS. Response rates and nonresponse errors in surveys. JAMA. 2012;307(17):1805-1806.)

**- The limitations section should be expanded to provide a more comprehensive discussion of the study's constraints. Specifically, it should be noted that the cross-sectional design limits any causal inferences, as it only allows for an association between variables to be observed at a single point in time. Additionally, the response rate of 24.1% introduces the possibility of selection bias, which could affect the generalizability of the results to the entire population of hospitalists in Korea. Including these points would help clarify the scope and limitations of the findings.**

Response: We appreciate your suggestion. We expanded and revised the limitation section as you recommended.

<Discussion, Sixth paragraph>

From: A limitation of this study is its reliance on a cross-sectional design coupled with an overall response rate of 24.1%, which is relatively low. Considering these aspects, it may be challenging to assert that this study comprehensively captured the situation of all Korean hospitalists. Nonetheless, the significance of this study stems from its distinction as the first to examine burnout and psychiatric symptoms among Korean hospitalists.

To: There are some limitations in this study. First, the design of this study is cross-sectional, which makes it difficult to determine temporal sequences between variables. Second, the overall response rate of 24.1% cannot entirely rule out the possibility of selection bias, which may affect the generalizability of the results to the entire population of Korean hospitalists. Considering these aspects, it may be challenging to assert that this study comprehensively captured the situation of burnout of all Korean hospitalists. Nonetheless, the significance of this study stems from its distinction as the first to examine burnout and psychiatric symptoms among Korean hospitalists.
