# Supplementary material for: Prevalence, risk and protective factors of burnout among Korean hospitalists
Source: PLoS One. 2025 Apr 28;20(4):e0320128. doi: 10.1371/journal.pone.0320128 (PMC12036936; doi:10.1371/journal.pone.0320128)
Supplement: Attachment — Submitted filename: reviewer comment_2nd revision_final.docx [file pone.0320128.s008.docx]

**Reviewer #2: I am happy that the authors made sufficient effort to address my comments. I still think the prevalence rate is a mistake and contributes to confusion on the topic - but they have explained this limitation.**

**The authors should however clearly state that the correlations between the components of burnout and DASS-depression were large, but well below cut-offs for discriminant validity issues. This adds to the literature.**

🡪 Thank you for clearly identifying important aspects that enhance the completeness of the study. According to previous research, if the correlation coefficient between different scales is less than 0.8, it can be judged that there is no issue with discriminant validity. As shown in Supplemental Table 1 of this study, the absolute values of the correlation coefficients between the components of Burnout and DASS-Depression are all below 0.6, confirming that there is no issue with discriminant validity. This information has also been incorporated into the manuscript.

From: More than half of the respondents screened positively for two burnout domains: 50.6% (n=40) for depersonalization, 57.0% (n=45) for reduced personal accomplishment, and 10.1% (n=8) reported high degree of emotional exhaustion (Figure 1). Of the respondents, 7.5% (n=6) reported mild to moderate depressive symptoms and 5.1% (n=4) reported mild to moderate anxiety symptoms. None of the respondents screened positive for severe depression, anxiety, or stress. Approximately 30.4% (n=24) of the respondents reported experiencing subthreshold insomnia and 8.9% (n=7) were screened for moderate to severe insomnia (Table 2). An analysis of the correlations between each burnout component, DASS, and ISI revealed statistically significant correlations, with the exception of the correlation between Reduced Personal Accomplishment and ISI (Supplemental Table 1).

To: More than half of the respondents screened positively for two burnout domains: 50.6% (n=40) for depersonalization, 57.0% (n=45) for reduced personal accomplishment, and 10.1% (n=8) reported high degree of emotional exhaustion (Figure 1). Of the respondents, 7.5% (n=6) reported mild to moderate depressive symptoms and 5.1% (n=4) reported mild to moderate anxiety symptoms. None of the respondents screened positive for severe depression, anxiety, or stress. Approximately 30.4% (n=24) of the respondents reported experiencing subthreshold insomnia and 8.9% (n=7) were screened for moderate to severe insomnia (Table 2). The correlation analysis between each burnout component, DASS, and ISI demonstrated statistically significant associations, except for the correlation between Reduced Personal Accomplishment and ISI. The absolute values of the correlation coefficients between the components of Burnout and DASS-Depression were all below 0.6, indicating no concerns regarding discriminant validity (Supplemental Table 2).[26]

[26] Clark, L.A., & Watson D. Constructing validity: Basic issues in objective scale development. Psychological Assessment. 1995;7(3):309-319. doi:10.1037/1040-3590.7.3.309.

**Reviewer #3: Thank you for the opportunity to review this interesting work. I was added as a reviewer after the first revisions, so I am reviewing the revised manuscript and appreciate the work of the authors and the reviewers in the improvements that are already evident. In summary, I find the revised manuscript appropriate for publication, and my comments are more on the order of suggestions and thoughts than requests for further revision:**

**-Beginning in the abstract but continuing throughout, I found the phrase "maximum number of inpatients" difficult to parse. I suspect it refers to a cap on the number of patients a hospitalist could manage in a day, but it almost reads as if burnout were decreased if the hospitalist managed the maximum number of patients possible. Some sort of clarification or rewording would be helpful to the reader.**

🡪 Thank you for your comments. As you mentioned, the term "maximum number of inpatients" does seem potentially confusing. Our intention was that a higher number of inpatients was associated with reduced burnout of hospitalists. As you pointed out, this might be not an intuitive finding. Our interpretation of these results is as follows; significant association between maximum number of inpatients and reduced burnout was only observed with reduced personal accomplishment subdomain, not in depersonalization or emotional exhaustion. This finding might suggest that when the inpatient load per hospitalist is too low, it might negatively impact their sense of professional satisfaction. This is an area that warrants further exploration in future research. We have addressed this point in the discussion as follows: "Additionally, a higher number of inpatients load per hospitalist was correlated with reduced burnout, specifically contributing to an increase in personal accomplishment within burnout domains. Considering the relatively short history of the domestic hospitalist system, it can be interpreted that achieving personal accommodation is facilitated by direct patient care, autonomous treatment, and appropriate workload allocation. However, it is worth noting that contrary to the general expectation that higher workload, which might relate to higher patient volumes, could worsen burnout. Further research is needed to explore this discrepancy." To minimize confusion, we will revise the term "maximum number of inpatients" to "cap on daily inpatient load per hospitalist."

From: “More than half of the respondents reported high graded burnout for two domains: depersonalization (50.6%) and reduced personal accomplishment (57%). Conflicts with caregivers, excessive workload, and long working hours were common risk factors for both burnout domains. The satisfaction with nonclinical work was identified as protective factors in depersonalization, and the availability of a research mentor and maximum number of inpatients were protective factors in reduced personal accomplishment. In the correlation analysis, the maximum number of inpatients and hospitalists per hospital was a significant factor in reducing burnout.”

To: “More than half of the respondents reported high graded burnout for two domains: depersonalization (50.6%) and reduced personal accomplishment (57%). Conflicts with caregivers, excessive workload, and long working hours were common risk factors for both burnout domains. The satisfaction with nonclinical work was identified as protective factors in depersonalization, and the availability of a research mentor and cap on daily inpatient load per hospitalist were protective factors in reduced personal accomplishment. In the correlation analysis, the maximum number of inpatients and hospitalists per hospital was a significant factor in reducing burnout.”

* All instances of "maximum number of inpatients" throughout the manuscript have been replaced with "cap on daily inpatient load per hospitalist."

**-In the Conclusions section of the abstract, the phrase “depersonalization and reduced personal accomplishments domain” seems like it should be “depersonalization and reduced personal accomplishment domains,” at least if I’m understanding it correctly.**

🡪 We agree with the reviewer's comment. The phrase should indeed be corrected to "depersonalization and reduced personal accomplishment domains" since it refers to two distinct domains of burnout. We will make this grammatical correction in the revised manuscript.

**-At least on the version I received, the abstract ends abruptly (and without punctuation), suggesting at least one missing word.**

🡪 We appreciate the reviewer pointing this out. Indeed, the abstract ended abruptly due to the omission of "quality of medical care" in the final sentence. We have now revised the abstract to include this missing phrase and added appropriate punctuation to ensure a complete and proper conclusion.

“Conclusions: This study revealed a high graded burnout rate of more than 50% in depersonalization and reduced personal accomplishments domain among Korean hospitalists, and found the risk and protective factors against burnout. The development of targeted interventions to mitigate burnout based on this study could enhance the mental well-being of healthcare professionals and improve the overall quality of medical care.”

**-It is an unfortunate consequence of the opposite orientation of the PA component of the MBI (relative to the EE and DP components) that discussing its results can be confusing. On page 8, the wording makes it sound like having a research mentor actually lowered the subjects' sense of personal accomplishment, when the Discussion makes clear that the opposite (the expected result) is true.**

🡪 We agree with the reviewer's point about the potential confusion in interpreting the Personal Accomplishment (PA) component of the MBI, which is scored in the opposite direction from the Emotional Exhaustion (EE) and Depersonalization (DP) components. We have revised the text to clarify that research mentorship is actually associated with a higher sense of personal accomplishment.

From: “The maximum number of inpatients and the availability of research mentors were significantly related to low total burnout scores and reduced personal accomplishment, while satisfaction with non-clinical work was significantly related to low total burnout scores and low depersonalization scores (Table 3).”

To: “The maximum number of inpatients and the availability of research mentors were significantly associated with low total burnout scores and higher sense of personal accomplishment, while satisfaction with non-clinical work was significantly related to low total burnout scores and low depersonalization scores (Table 3).”

**-The sentence at the bottom of page 10 (carrying over to page 11) does not make sense and appears to be missing a clause. It sets up a "contrary to" that never resolves, at least in my copy.**

🡪 We thank the reviewer for pointing out the incomplete sentence structure. We have revised the text to complete the "contrary to" comparison by adding the resolution clause. The revised version now properly presents the contrast between the general expectation about workload and burnout, and our study findings.

From: “Considering the relatively short history of the domestic hospitalist system, it can be interpreted that achieving personal accommodation is facilitated by direct patient care, autonomous treatment, and appropriate workload allocation. However, it is worth noting that contrary to the general expectation that higher workload, which might relate to higher patient volumes, could worsen burnout. Further research is needed to explore this discrepancy.”

To: "Considering the relatively short history of the domestic hospitalist system, it can be interpreted that achieving personal accommodation is facilitated by direct patient care, autonomous treatment, and appropriate workload allocation. Contrary to the general expectation that higher workload and higher patient volumes could worsen burnout, our findings suggest a different pattern. Further research is needed to explore this discrepancy."

**-I understand the intended meaning, but on page 11 I would avoid the phrase "negative marital status" to refer to single physicians, using "single" or "unmarried" instead. I work with a number of unmarried physicians who consider it quite a positive status!**

🡪 We appreciate the reviewer's thoughtful feedback regarding the terminology. We agree that the phrase "negative marital status" could imply an unintended value judgment. We have revised the text to use "unmarried" instead, which is a more neutral and appropriate term to describe marital status.

From: “Certain demographic factors, including young age, female sex, and negative marital status are also risk factors for vulnerability to burnout.”

To: “Certain demographic factors, including young age, female sex, and unmarried are also risk factors for vulnerability to burnout.”

**-In a bigger picture sense, I'm fascinated by the aspect of this data that it reflects the experiences of a country introducing hospitalists long after many other countries did so. Although the history of this development is nicely laid out in the Introduction, I feel a paragraph in the Discussion about the usefulness of this data to other countries considering using the hospitalist model could make the data even more compelling than the novelty of the first report of Korean hospitalist burnout already does.**

🡪 Thank you for your novel suggestion. We added about the usefulness of our data to other countries using the hospitalist models in the discussion.

From: “Nonetheless, the significance of this study stems from its distinction as the first to examine burnout and psychiatric symptoms among hospitalists.”

To: “Nonetheless, the significance of this study stems from its distinction as the first to examine burnout and psychiatric symptoms among hospitalists during the early adoption phase of the hospitalist system in South Korea. Unlike the United States, where the system has been established for over 20 years and is relatively well-defined, this study explores the experience of South Korea, where the hospitalist system is still in its early stages of implementation. The findings of this study hold significance as they may serve as a valuable reference for other countries that are considering implementing the hospitalist system or are in the initial phase of its adoption.”
